# Biomechanical comparison of canine femurs implanted with either cemented (CFX^®^) or cementless (with lateral bolt) (BFX^®^+lb) total hip replacement under 4-point bending or torsional loads

**DOI:** 10.3389/fbioe.2023.999271

**Published:** 2023-03-08

**Authors:** Selena Tinga, David D. Tuyn, Rosalind J. Kopp, Stanley E. Kim

**Affiliations:** ^1^ Comparative Orthopaedics and Biomechanics Laboratory, College of Veterinary Medicine, University of Florida, Gainesville, FL, United States; ^2^ Department of Mechanical and Aerospace Engineering, University of Florida, Gainesville, FL, United States

**Keywords:** total hip replacement, CFX, BFX, lateral bolt, 4-point bending, axial torsion

## Abstract

**Objective:** Compare biomechanical properties of femurs implanted with either BioMedtrix^™^ biological fixation with interlocking lateral bolt (BFX^®^+lb) or cemented (CFX^®^) stems when subjected to 4-point bending or axial torsional forces.

**Study Design:** Twelve pairs of normal medium to large cadaveric canine femora were implanted with a BFX + lb (*n* = 12) and a CFX (*n* = 12) stem–one in the right and one in the left femora of the pair. Pre- and post-operative radiographs were made. Femora were tested to failure in either 4-point bending (*n* = 6 pairs) or axial torsion (*n* = 6 pairs), and stiffness, load or torque at failure, linear or angular displacement, and fracture configuration were noted.

**Results:** Implant position was acceptable in all included femora, but CFX stems were placed in less anteversion than BFX + lb stems in the 4-point bending group (median (range) 5.8 (−1.9–16.3) vs. 15.9 (8.4–27.9) anteversion, respectively (*p* = 0.04)). CFX implanted femora were more stiff than BFX + lb implanted femora in axial torsion (median (range) 2,387 (1,659–3,068) vs. 1,192 (795–2,150) N*mm/^o^, respectively (*p* = 0.03)). One of each stem type, from different pairs, did not fail in axial torsion. There was no difference in stiffness or load to failure in 4-point bending, or in fracture configuration for either test, between implant groups.

**Conclusion:** Increased stiffness of CFX implanted femurs under axial torsional forces may not be clinically relevant as both groups withstood expected *in vivo* forces. Based on this acute post-operative model using isolated forces, BFX + lb stems may be a suitable replacement for CFX stems in femurs with normal morphology (stovepipe and champagne flute morphology were not tested).

## Introduction

Canine hip dysplasia (CHD) is a common orthopedic condition in dogs (Johnson et al., 1994). There are several management strategies for CHD, including medical management or various surgical procedures. Total hip replacement (THR) is applied to relieve pain after failure of medical management, with the goal of restoring normal biomechanics and kinematics (Kowaleski et al., 2013).

Originally, THR was performed by cementing implants into prepared cavities in the femur and acetabulum (Hoefle, 1974; Olmstead et al., 1983). A major long-term complication of cemented THR is aseptic loosening, present in up to 63% of cases and creating a clinical problem for the animal in up to 7% of cases (Olmstead, 1987; Edwards et al., 1997; Skurla et al., 2005; Bergh et al., 2006). Cementless implants were developed in part to overcome aseptic loosening (Schiller, 2017). With most cementless implants, initial strength relies on a press-fit mechanism and osteointegration occurs over time to strengthen the bone-implant interface (Schiller, 2017), therefore the long-term risk of implant loosening is reduced compared to cemented systems (Marcellin-Little et al., 1999). Despite this, cemented femoral implants are still used commonly. For example, many surgeons will choose a cemented stem for dogs with a femoral canal flare index (CFI) ≤ 1.8 (“stovepipe” morphology) because initial press-fit may not be achieved with cementless implants in these femurs. Without press-fit, stem subsidence, retroversion, or micromotion may occur which may lead to coxofemoral luxation, femoral fracture, failure of osteointegration, and poor outcome, often requiring revision surgery (Engh et al., 1992; Rashmir-Raven et al., 1992; Ganz et al., 2010; Schiller, 2017).

For dogs, both cemented (CFX^®^, BioMedtrix^™^ (BioMedtrix, Boonton, NJ)) and cementless (BFX^®^, BioMedtrix^™^ (BioMedtrix, Boonton, NJ)) total hip replacement implant systems are available (Schiller, 2017). Stem options within the BFX system include a standard stem, a collared stem, and more recently a stem with the option to place an interlocking lateral bolt (BFX + lb) (Schiller, 2017). Initial stability of all BFX stems rely on a press-fit mechanism and all have a partially porous surface to encourage bone ingrowth (Schiller, 2017). In addition to press-fit, the collared stem resists subsidence if the collar rests on the osteotomy and the BFX + lb stem resists subsidence and axial rotation *via* the lateral bolt prior to osteointegration (Schiller, 2017). The lateral bolt is placed through a guided drill hole in the lateral aspect of the proximal femur, screws into the lateral aspect of the BFX + lb stem, and protrudes through the lateral cortex (Schiller, 2017). The BFX + lb stem has been shown to prevent subsidence when compared to both traditional BFX stems in a cadaveric biomechanical study (Buks et al., 2016) and collared BFX stems in a retrospective clinical radiographic study (Mitchell et al., 2020) supporting the use of BFX + lb stems in place of traditional and collared stems for prevention of subsidence. Consequently, the BFX + lb stem is purported to be applicable to stovepipe shaped femurs (where previously a CFX stem would have been used and long-term complications such as aseptic loosening would be a concern) (Schiller, 2017). Importantly, the surgeon must make the final decision regarding stem type in the operating room, based on multiple factors other than radiographic femoral morphology and cortical appearance, such as cancellous bone quality, which is best assessed during broaching (Schiller, 2017).

While the BFX + lb and CFX stems appear to be interchangeable in many cases, there have not been any direct biomechanical comparisons of BFX + lb to CFX implanted femurs. Therefore, the purpose of the current study was to compare BFX + lb implanted femurs to CFX implanted femurs with regards to ultimate failure, stiffness, and fracture configuration when subjected to 4-point bending or axial torsional forces. We hypothesized that the CFX implanted femurs would 1) fail at higher loads and 2) have higher stiffness than the BFX + lb implanted femurs in both 4-point bending and axial torsional testing. We also hypothesized that 3) 4-point bending would induce a fracture at the distal aspect of the stem regardless of stem type, and 4) axial torsional loads would induce a spiral fracture that would originate from the osteotomy in CFX implanted femurs and from the bolt hole in BFX + lb implanted femurs.

## Methods

This study was approved by the University of Florida Institutional Animal Care and Use Committee. Dogs were euthanized for reasons unrelated to the study and donated to research purposes. Paired femora were harvested from adult medium to large dogs with normal to mildly dysplastic coxofemoral joints, and soft tissues were removed. Pairs were excluded if orthopedic disease beyond coxofemoral laxity or mild osteophytosis of the femoral head and/or acetabulum was present in the hip joint; no pairs were included with moderate to severe osteophytosis or moderate to severe femoral neck thickening/shortening. Orthogonal radiographs were made and radiographic skeletal maturity and bone health was confirmed–any pairs with open physes or bony lesions such as trauma or neoplasia were excluded. Stem size was estimated by calibrating the cranial-caudal radiograph and overlaying the BFX stem acetate templates of increasing sizes; the stem size that allowed a 1–2 mm gap between the stem and the cortex at the level of the porous-smooth junction and distally, with the shoulder of the stem at the level of the proximal 1/3 of the greater trochanter was chosen (Schiller, 2017). Only femora that were able to accept BFX + lb stem sizes 7–10 based on digital radiographic templating were included.

### Specimen preparation

After pre-operative radiographs, femora were wrapped in saline (0.9% NaCl) soaked gauze and stored at −5°C until implantation. Femora were thawed to room temperature overnight, then femoral canals were prepared and stems implanted as previously described (Schiller, 2017). For each pair of femora, one BFX + lb and one CFX implant was placed, alternating right and left femurs for each implant type. The BFX femur was prepared first, and stem sizing was confirmed during implantation based on achieving a press-fit feel during broaching and BFX + lb implantation. Any pair of femurs that was not able to accept a BFX + lb stem size 7–10 based on intraoperative feel was excluded even if previously included based on radiographic templating. The contralateral CFX femur was prepared in the same fashion, the femoral canal was flushed, and a CFX stem two sizes smaller than the corresponding BFX implant was used. Cement^a^ was mixed under vacuum^b^, a cement plug was inserted, a cement gun was used to deliver cement from distal to proximal with manual pressurization, and the stem (with centralizer) was guided into the femoral canal by hand and secured manually until the cement was set. Post-operative orthogonal radiographs were made and the femora were wrapped in saline (0.9% NaCl) soaked gauze and stored at −5°C until preparation for mechanical testing. The femora were thawed at room temperature overnight, then three screws (3.5 mm cortical screws) were placed in the distal metaphysis of each femur, which were left protruding to serve as anchors in the potting material^c^. Femora were potted at the distal end using uniform square cardboard molds such that the long axis of the femur was perpendicular to the bottom of the mold (perpendicularity was confirmed with orthogonal plumb lines). The proximal end was not potted. Potted femora were stored at 10 °C and mechanical testing was performed the following day to ensure that the potting material was fully set. A minimum of 2 days passed between stem implantation and biomechanical testing.

### Radiographic evaluation

**FIGURE 1:**
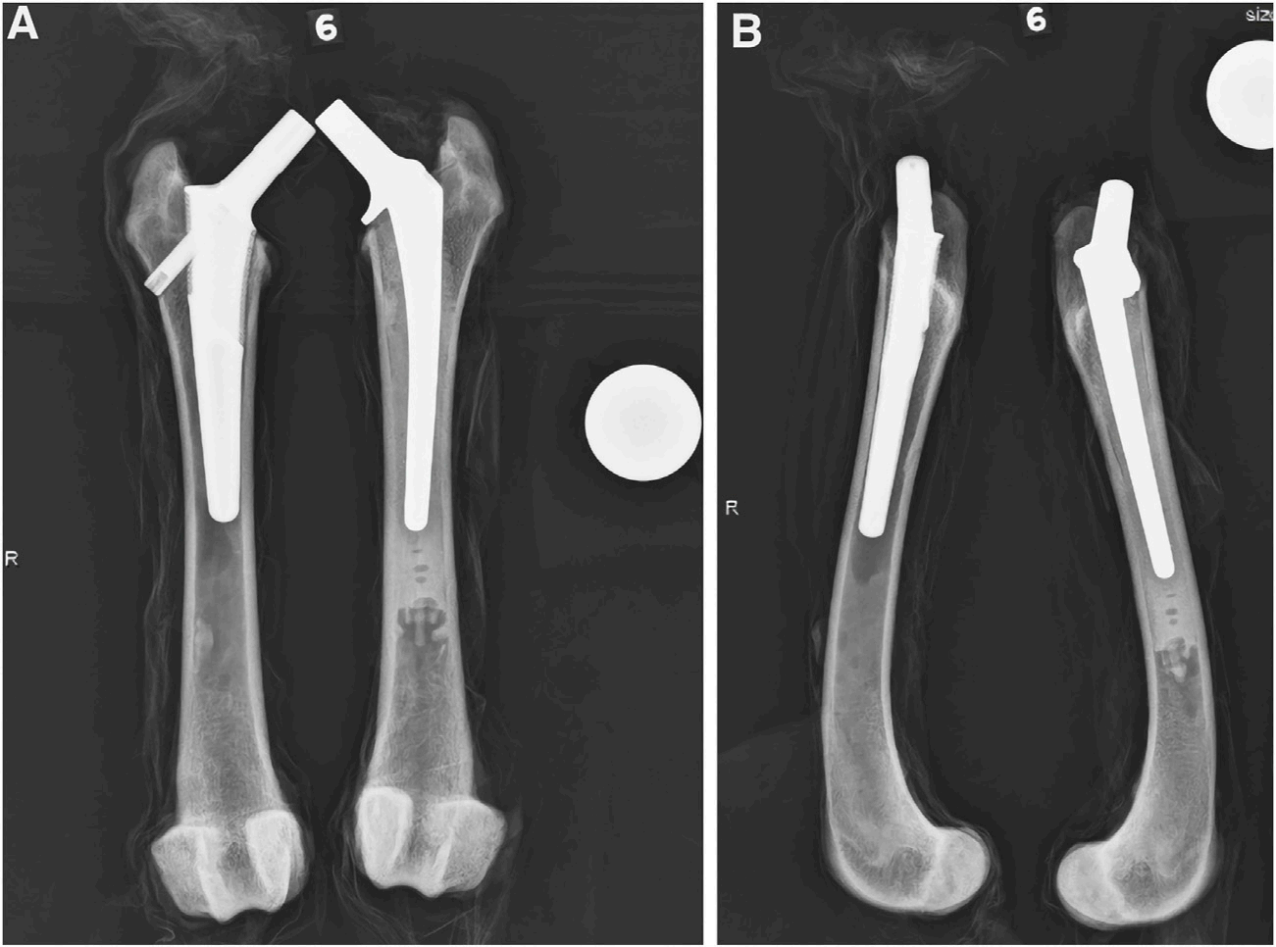
Representative cranial-caudal **(A)** and medial-lateral **(B)** radiographic views after stem implantation. The BFX + lb implanted femur is on the left and CFX implanted femur is on the right.

All measurements were made by one person (ST). Femoral length was measured from the proximal-most aspect of the greater trochanter to the distal-most aspect of the lateral condyle on the cranial-caudal radiographic projection. Femoral CFI was calculated as the ratio between canal width at the lesser trochanter and at the narrowest point of the isthmus and any femurs with CFI less than 1.8 (stovepipe morphology) or greater than 2.5 (champagne flute morphology) were excluded. Representative post-operative radiographs are shown in Figure 1. After BFX + lb implantation, canal fill was measured on the cranial-caudal and medial-lateral radiographic projections as previously described (DeYoung and Schiller, 1992; Townsend et al., 2016). After CFX implantation, the quality of the cement mantle was graded subjectively (A, B, C, or D as previously reported where A is complete filling of the medullary cavity below the lesser trochanter and no radiolucency at the bone-cement interface and D is radiolucency at 100% of the bone-cement interface or no cement past the tip of the stem) (Barrack et al., 1992; Ota et al., 2005). Stem orientation was evaluated on the cranial-caudal radiographic projection (varus/valgus) and on the medial-lateral radiographic projection (cranial/caudal tipping, calculated version angle). With regards to varus/valgus or cranial/caudal tipping, a neutral position indicates that the central axis of the stem is parallel to the central axis of the femur (DeYoung and Schiller, 1992). A varus stem position is defined as medial tipping of the stem with the distal tip of the stem deviated towards the lateral cortex, and *vice versa* for a valgus stem position (DeYoung and Schiller, 1992). A cranially tipped stem is defined as having the distal tip of the stem deviated towards the caudal cortex, and *vice versa* for a caudally tipped stem (DeYoung and Schiller, 1992). Prosthesis version angle was calculated from measurements taken on the medial-lateral radiograph using a trigonometric method modified from that published by Bausman and Wendelburg. (2013), but using two central points in the prosthesis neck to define the central neck axis (as prosthetic heads were not in place during radiographs).

### Mechanical testing

Custom fixtures were created for each test, for use in a servo-hydraulic materials testing (MTS) machine. ^d^ The MTS machine is equipped with an axial-torsional load cell and axial force and torque were measured with an axial torsional load transducer with 15 kN axial, 150 Nm torsional load limits. Each construct was tested to failure. A high-definition digital video camera^e^ was used to record fracture propagation, including the location of initiation of the fracture when possible. The cranial surface of the bone was facing the camera for 4-point bending tests and the lateral surface of the bone was facing the camera for torsional testing.

### 4-Point bending

**FIGURE 2:**
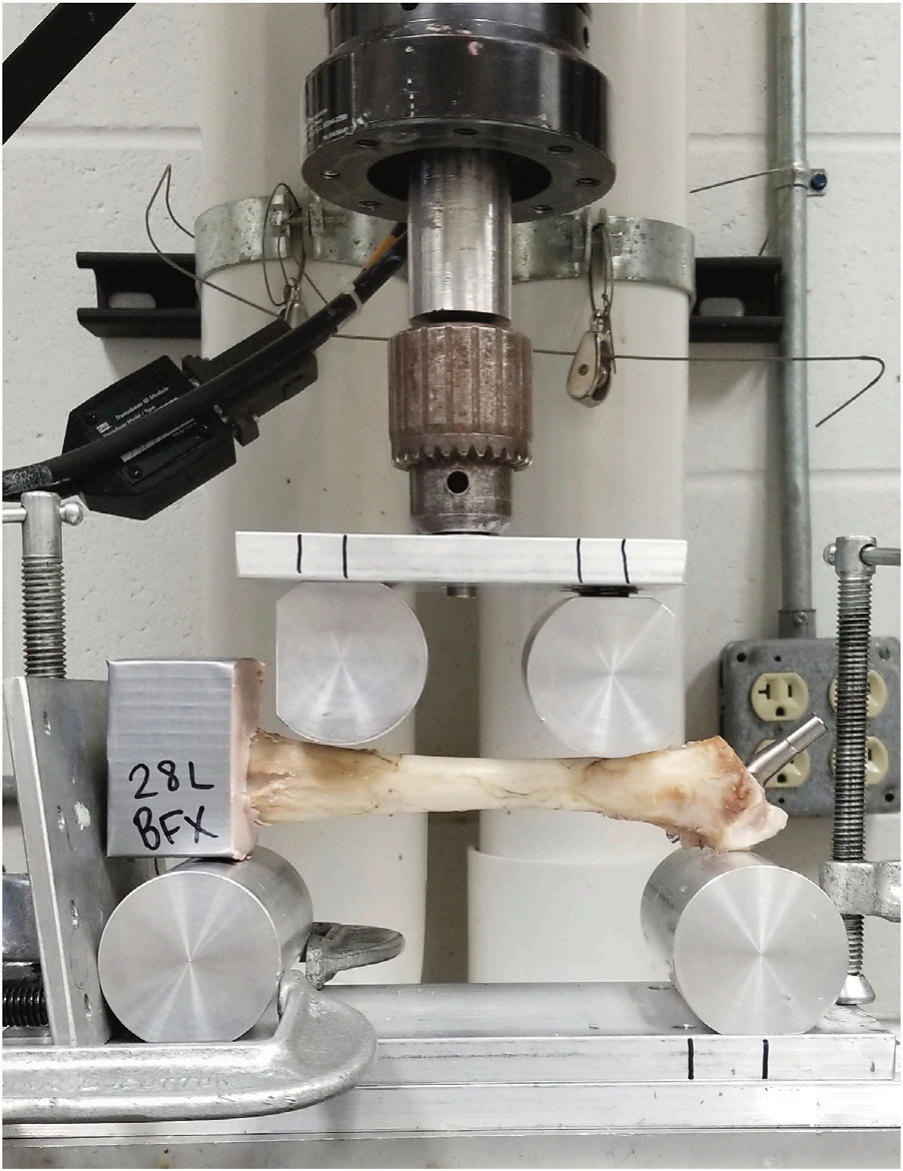
Four-point bending fixture loaded with potted left femur with implanted BFX + lb, prepared for testing in the MTS machine. The cranial aspect of the femur is facing the camera and the lateral surface of the femur faces down; the protruding portion of the lateral bolt can be seen just distal to the greater trochanter, near the proximal-most support roller.

The 4-point bending fixture was manufactured from 6061 T6 aluminum (Figure 2). The base of the fixture supported the lateral aspects of the bone using two support rollers fastened to a baseplate, with the spacing between the support rollers adjusted based on the distance between the greater trochanter and the lateral femoral epicondyle. The load was applied to the medial cortex through two load rollers, which were fastened to a plate (spaced at 50% that of the support rollers) that attached to the crosshead of the MTS. This spacing allowed for the entire bone implant interface to experience the largest bending load, while still accommodating the potted construct. The square shape of the potting mold prevented axial rotation of the bone during testing in order to limit the application of the bending force to the lateral (tension) and medial (compression) surfaces of the bone. Each specimen was preconditioned by placing the rollers at 50 N load then lifting the rollers 3 mm; this was repeated 5 times at 0.2 Hz. Then, the femur was loaded to failure at a rate of 0.4 mm/s.

### Axial torsion

**FIGURE 3:**
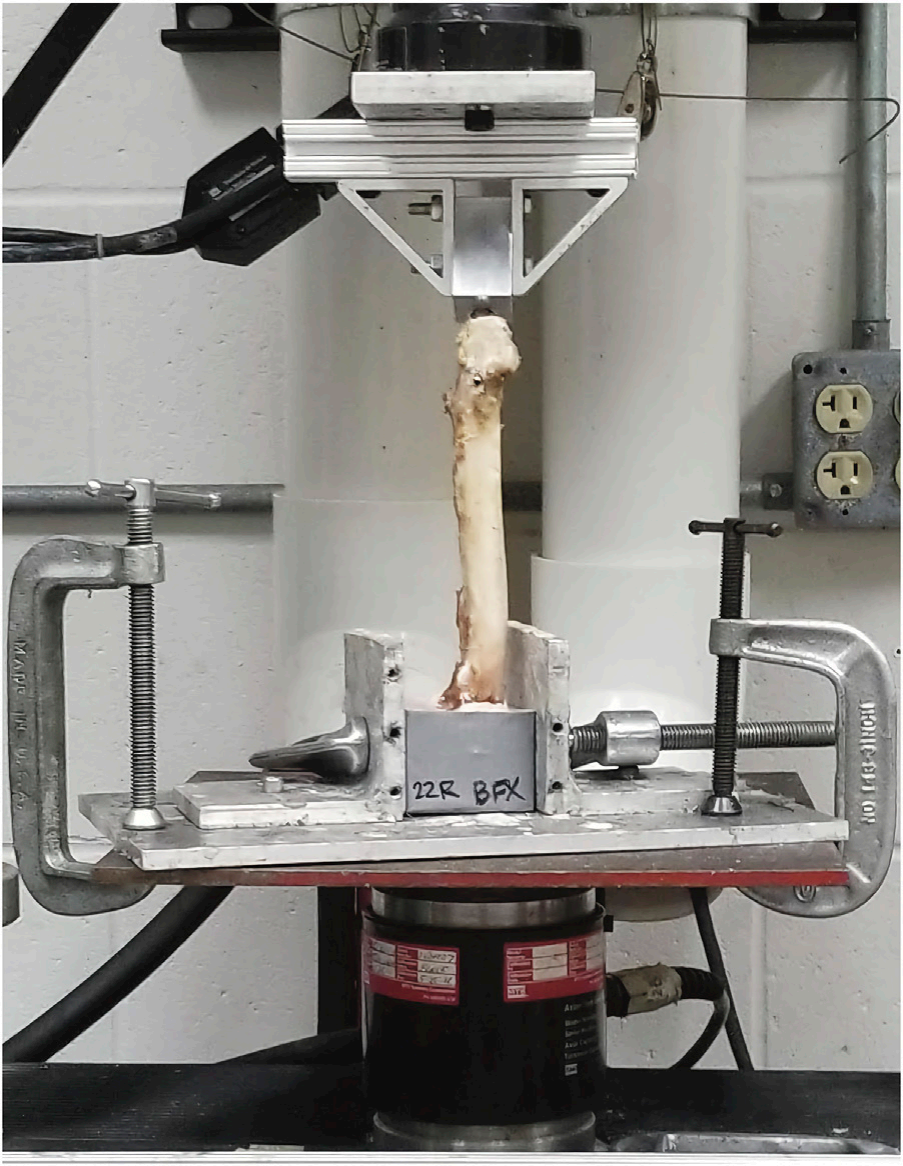
Axial torsion fixture loaded with potted right femur with implanted BFX + lb, prepared for testing in the MTS machine. The lateral aspect of the femur is facing the camera and the lateral protruding portion of the lateral bolt is visualized caudolaterally, just distal to the greater trochanter. The cranial aspect of the femur is to the right of the image.

The torsion fixture was designed based on previous studies using a machined aluminum block that stabilized the femoral head and neck and prevented slipping of the prosthesis during testing (Figure 3). (Pozzi et al., 2013; Christopher et al., 2016) The bone was aligned with the load actuator and secured into a square base to prevent rotation of the femur. Care was taken to ensure that each femoral construct was loaded into the MTS machine in a neutral position (the neck of the implant was central within the trough of the fixture) so that no unintended rotational or other forces were applied. Each specimen was preconditioned by axial compressive cycling between 30 N and 300 N at 1 Hz for 30 cycles. After preconditioning, the specimens were loaded to 300 N axial compression, then internal rotation (retroversion) was applied to the implant at a rate of 10°/s to a maximum of 45. The direction of force (internal rotation/retroversion) was selected to mimic the more common direction of rotational failure in uncemented constructs (Schiller, 2017). The degrees at ultimate failure was corrected to account for an initial toe region of the data that implied a delay in the fixture contacting the neck of the implant.

### Fracture configuration

Fractures were categorized by examination immediately after testing, palpation of stem motion, and review of the testing video in slow motion. Post-testing photographs were obtained of the cranial, medial, caudal, and lateral bone surfaces. Fractures were categorized based on the Vancouver Classification (Table 1). (Brady et al., 2000; Liska, 2004; Christopher et al., 2016)

**TABLE 1:** Vancouver classification of femoral fractures after total hip replacement.

Class	Description
A	Involves the greater or lesser trochanters but not the diaphysis
B1	Involves the proximal diaphysis to the level of the distal stem or cement mantle; stable prosthesis
B2	Involves the proximal diaphysis to the level of the distal stem or cement mantle; unstable prosthesis with adequate bone stock for revision
B3	Involves the proximal diaphysis to the level of the distal stem or cement mantle; unstable prosthesis and catastrophic bone loss
C	Involves the distal diaphysis

### Data analysis and statistical comparisons

For 4-point bending, stiffness was calculated based on the first 100 N of the linear portion of the load/displacement curve (a standardized portion of the curve prior to elastic deformation). Load (N) and displacement (mm) at failure were defined as the load and linear displacement at which the fracture was first initiated and was associated with a sudden decrease in load. Stiffness and load at failure were subjectively compared between grades of cement mantles in CFX implanted femora.

For axial torsion, stiffness was calculated based on the first 5000 N*mm of the linear portion of the load/displacement curve (a standardized portion of the curve prior to elastic deformation). Torque (N*mm) and angular displacement (o) failure were defined as the torque and displacement at which fracture was first initiated and was associated with a sudden decrease in torque. Stiffness and torque at failure were subjectively compared between grades of cement mantles in CFX implanted femora.

Non-parametric tests were used to avoid assumption of normality in statistical calculations, due to small sample size. Femoral morphology, stiffness, load at failure, and displacement at failure were compared between BFX + lb and CFX implanted paired femora using a related samples Wilcoxon signed rank test, and implant position was compared using a Wilcoxon signed rank test (SPSS). *p* ≤ 0.05 was considered statistically significant.

## Results

Twelve pairs of femora were distributed into testing groups for inclusion in the final data set: six pairs were tested in 4-point bending and six pairs were tested in torsion (Table 2). Femoral CFI had a median (range) of 2.06 (1.87–2.17) for the BFX + lb femurs and 2.10 (1.97–2.24) for the CFX femurs for specimens tested in 4-point bending, and 1.94 (1.83–2.04) for the BFX + lb femurs and 1.93 (1.81–2.07) for the CFX femurs for specimens tested in axial torsion. All BFX + lb implanted femora had acceptable canal fill. Femora tested in 4-point bending included implant sizes BFX + lb 7–10 (CFX 5–8) and femora tested in axial torsion included implant sizes BFX + lb 7–9 (CFX 5–7). For the sizes tested in axial torsion, the neck length for CFX implanted femora was 2 mm shorter than the paired BFX + lb implanted femora as per implant specifications. Implant position was considered acceptable in all cases; however, in the 4-point bending group, femora implanted with CFX stems had less anteversion than femora implanted with BFX + lb stems.

**TABLE 2:** Femoral length and canal flare index, and radiographic analysis of THR implantation in femora tested in 4-point bending (top) and in axial torsion (bottom). Reported values are median (range). A positive stem orientation in the frontal plane indicates stem varus, a positive stem orientation in the sagittal plan indicates cranial tipping, and a positive version indicates anteversion. Femoral length and canal flare index were compared using a related-samples Wilcoxon signed rank test. All other measurements were compared using a Wilcoxon signed rank test. *p* < 0.05 was considered statistically significant. Cemented stems were placed in less anteversion than BFX + lb stems for the group tested in 4-point bending.

4-Point bending			
	BFX + lb (n = 6)	CFX (n = 6)	*p*-value
Femoral length (mm)	186.6 (171.5–203.3)	188.9 (173.1–200.6)	0.23
Femoral canal flare index	2.06 (1.87–2.17)	2.10 (1.97–2.24)	0.35
Femoral canal fill (frontal) (%)	75.0 (71.9–78.2)	n/a	n/a
Femoral canal fill (sagittal) (%)	72.7 (66.5–78.7)	n/a	n/a
Stem orientation (frontal) (^o^)	−1.0 (−1.1–2.9)	−1.3 (−3.2–1.2)	0.17
Stem orientation (sagittal) (^o^)	0.4 (−1.5–2.3)	0.6 (−0.9–1)	0.50
Stem version (^o^)	15.9 (8.4–27.9)	5.8 (−1.9–16.3)	**0.04**

Axial torsion			
	BFX + lb (n = 6)	CFX (n = 6)	*p*-value
Femoral length (mm)	167.2 (157.3–196.9)	166.7 (157.5–197.0)	0.46
Femoral canal flare index	1.94 (1.83–2.04)	1.93 (1.81–2.07)	0.92
Femoral canal fill (frontal) (%)	70.1 (67.3–72.1)	n/a	n/a
Femoral canal fill (sagittal) (%)	67.3 (61.8–82.8)	n/a	n/a
Stem orientation (frontal) ^o^)	0 (−2.6–0.6)	−1.3 (−1.8–0.4)	0.46
Stem orientation (sagittal) ^o^)	1.4 (−2.1–2.7)	1.2 (−0.4–4.8)	0.46
Stem version ^o^)	18.3 (12.5–24.9)	5.8 (1.5–25.3)	0.12

### 4-Point bending

**FIGURE 4:**
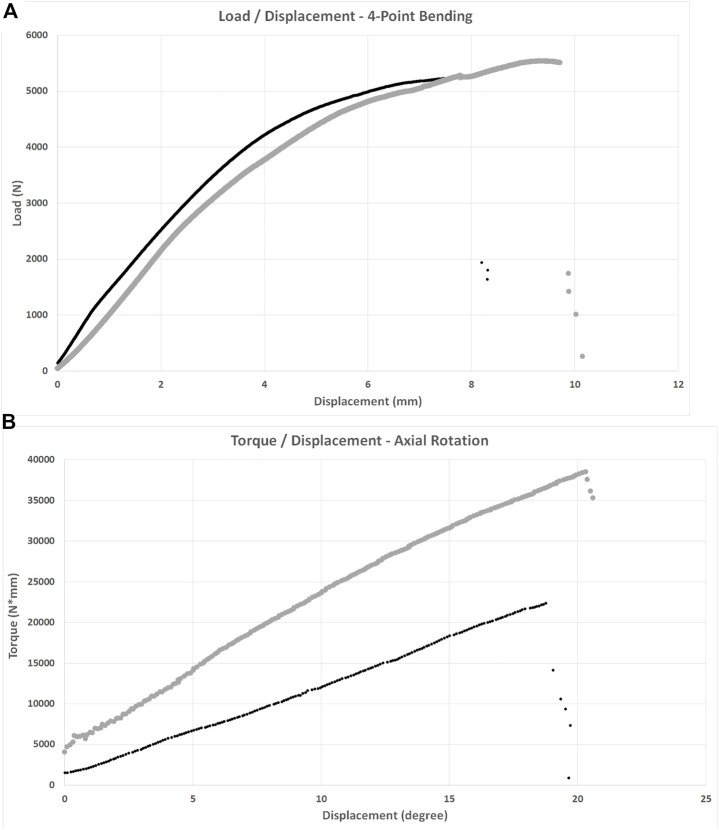
Load/displacement curves for 4-point bending **(A)** and axial torsion **(B)** BFX + lb implanted femurs are indicated using black markers and CFX implanted femurs are indicated using grey markers. For axial torsion testing, the degrees at ultimate failure was corrected to account for an initial toe region of the data that implied a delay in the fixture contacting the neck of the implant. For both tests, stiffness was calculated from the initial linear portion of each curve and ultimate failure was defined as the point at which load acutely decreased. CFX implanted femurs were stiffer than BFX + lb implanted femurs when subjected to axial torsion.

There was no difference in stiffness, force to ultimate failure, or displacement at ultimate failure between implant systems (Table 3). Sample load/displacement curves from a single pair of femora subjected to 4-point bending are displayed in Figure 4A.

**TABLE 3:** Results of 4-point bending test (top) and axial torsion test (bottom). Reported values are median (range). The degrees at ultimate failure was corrected to account for an initial toe region of the data that implied a delay in the fixture contacting the neck of the implant. Measurements were compared using a Wilcoxon signed rank test. *p* < 0.05 was considered statistically significant. There was no difference in stiffness or failure between femora implanted with BFX + lb or CFX when subjected to 4-point bending, but femora implanted with CFX were stiffer than femora implanted with BFX + lb when subjected to axial torsion. **Only 5/6 femurs from each implant group (different pairs) failed during torsional testing so the reported *p*-values for load and displacement at failure was calculated using *n* = 4 pairs.

4-Point bending			
	BFX + lb (n = 6)	CFX (n = 6)	*p*-value
Stiffness (N/mm)	966 (664–1,426)	953 (647–1,044)	0.25
Load at ultimate failure (N)	4,284 (3,605–5,254)	4,188 (3,157–5,506)	0.75
Displacement at ultimate failure (mm)	7.0 (4.9–11.5)	8.6 (4.5–11.3)	0.17

Axial torsion			
	BFX + lb (n = 6) **	CFX (n = 6) **	*p*-value
Stiffness (N*mm/^o^)	1,192 (795–2,150)	2,387 (1,659–3,068)	**0.03**
Torque at ultimate failure (N*mm) **	25,930 (19,778–40,919)	38,503 (21,505–53,969)	0.14
	*1 did not fail	*1 did not fail	
Displacement at ultimate failure (^o^) (corrected) **	19.6 (14.3–34.3)	23.3 (12.6–29.2)	1.00
	*1 did not fail	*1 did not fail	

The fracture pattern was variable. In BFX + lb implanted femora, three were Vancouver class B1 (transverse or short oblique fractures with mild comminution at level of the distal stem), two were Vancouver class B2 (spiral fractures originating from the bolt hole) and one was a Vancouver class C fracture. The fracture pattern for CFX implanted femora was more consistent: five were Vancouver class B1 (transverse to short oblique or spiral with minimal comminution at level of distal stem or cement mantle) and one was a Vancouver class B2 fracture (medial and lateral longitudinal fractures originating from osteotomy).

### Axial torsion

Femora implanted by CFX were stiffer in torsion compared to femora implanted by BFX + lb (Table 3). There was no difference in torque to ultimate failure or degrees of rotation (corrected) at ultimate failure between implant systems (Table 3). Sample torque/displacement curves from a single pair of femora subjected to 4-point bending are displayed in Figure 4B. One femur from each treatment group did not fail; these were from different pairs.

The fracture pattern was less variable in femora that failed in axial torsion (Five BFX + lb and five CFX implanted femurs). In BFX + lb implanted femora, two were Vancouver class B1 and three were Vancouver class B2 fractures. All were spiral fractures; two originated from the bolt hole and from the medial aspect of the osteotomy and three originated from just the medial aspect of the osteotomy with the bolt hole remaining intact. In CFX implanted femora, two were Vancouver class B1 and three were Vancouver class B2 fractures (loose at stem-cement interface). All were spiral fractures originating from the osteotomy.

### Cement mantle quality

For CFX implanted femora, cement mantle varied from grade A to C with most having grade B cement mantle quality (Table 4). When stiffness or load at ultimate failure was detailed for femurs with various grades of cement mantle quality, there did not appear to be any association between cement mantle quality and outcome, but sample sizes did not allow for statistical comparison.

**TABLE 4:** Cement mantle grade, where A indicates complete filling of the medullary cavity by cement below the lesser trochanter with no radiolucency at the bone-cement interface, B indicates radiolucency up to 50% of bone-cement interface, C indicates radiolucency at 50%–99% of the bone-cement interface or defective/incomplete cement mantle, and D indicates radiolucency at 100% of the bone-cement interface or no cement past the tip of the femoral component. No CFX stems had grade C or D cement mantles in the 4-point bending group and no CFX stems had grade A or D cement mantles in the axial torsion group. Cement mantle grade did not appear to affect stiffness or load at failure. *Only 3/4 CFX implanted femurs with cement mantle grade B failed during torsional testing so the reported ultimate failure has *n* = 3.

4-Point bending			
Grade	A	B	C
Number	2	4	0
Stiffness (N/mm)	981 (784–995)	914 (647–1,044)	n/a
Load at ultimate failure N)	4,390 (3,157–5,408)	4,284 (4,049–5,506)	n/a

Axial torsion			
Grade	A	B	C
Number	0	4 **	2
Stiffness (N*mm/^o^) **	n/a	2,387 (1,679–2,949)	2,364 (1,659–3,068)
Torque at ultimate failure (N*mm) **	n/a	38,503 (21,505–49,611)	44,187 (34,404–53,969)

## Discussion

The BFX + lb stem was developed, in part, to replace the need for the use of the CFX femoral system in some situations such as a stovepipe femoral morphology (Schiller, 2017). Direct comparison between the two systems is important to assure that use of a BFX + lb stem in place of a CFX stem will not result in an increase in complications such as increased incidence of femoral fracture or a more catastrophic fracture configuration when fracture occurs. In this cadaveric biomechanical study, stiffness, load and displacement at ultimate failure, and fracture configuration, were compared between BFX + lb and CFX implanted cadaveric femora subjected to either 4-point bending or axial torsional loads. We rejected our first, third, and fourth hypotheses: there was no difference in ultimate failure between BFX + lb and CFX implanted femurs under either 4-point bending or axial torsional loads and the fracture configurations did not follow a distinct pattern other than to fall generally into the Vancouver class B category. We accepted our second hypothesis as it related to axial torsional testing: the CFX implanted femora were stiffer compared to the BFX + lb implanted femora when tested in axial torsion.

When subjected to axial torsion, CFX implanted femora were approximately twice as stiff as BFX + lb implanted femora. The median torque at failure in the BFX + lb implanted femora (25,930 N*mm) was similar to slightly higher than previously reported values for standard BFX stems (18,980 N*mm) (Christopher et al., 2016) or Zurich Cementless stems (20,900–21,900 N*mm) (Pozzi et al., 2013) without adjunctive fixation. In the acute post-operative stage, our finding that CFX implanted femora are stiffer than BFX + lb implanted femora when subjected to torsional forces is likely to be consistent with the *in vivo* situation where the cement mantle surrounding the CFX implant is at its strongest (the stem-cement interface has not yet started to loosen) but the press-fit BFX + lb implanted femur is at its weakest (the implant is not yet osteointegrated). Proper cementing technique will force the cement into the interdigitations of the cancellous bone (Schiller, 2017), and the cement forms a rigid customized envelope around the stem, so as the torsional force was applied to the implant head it was translated to the cortical bone which twisted until its elastic capacity was exceeded and fracture occurred. The stiffness of the CFX implanted femora may have been different if the cement mantles had been grade A or grade D in quality. While our test is replicating the acute post-operative situation only, it is interesting to note that previous studies have shown that femoral cement mantle grade does not correlate with *in vivo* stem loosening in studies of human or canine patients, which may be due to other surgical factors such as advancing stem design and use of a cement plug to improve pressurization which may improve the functional quality of the cement mantle in ways that may not be entirely represented by radiographic grading (Barrack et al., 1992; Ota et al., 2005). In addition to surgical technique, many patient and owner-related factors are likely to influence CFX stem loosening *in vivo*. In contrast to CFX, a BFX + lb construct will not achieve implant-bone rigidity until osteointegration occurs. Therefore, as torsion was applied to the BFX + lb implanted femora in this acute scenario, the implant shifted within the medullary cavity with less force. However, the difference in stiffness may not be clinically relevant as both treatment groups were able to withstand torsional forces that were much higher than the highest torsional load that has been identified *in vivo* in the canine hip: approximately 1,600 N*mm during walking (Page et al., 1993). Alternatively, given that patients would be exposed to a high number of cyclic loads rather than an acute load, the difference in stiffness could be relevant particularly during the time before osteointegration of the BFX + lb implant. It is also important to consider the effect of neck length on torsional stiffness–a longer neck will result in a reduction in torsional stiffness. While neck length is determined intra-operatively during hip reduction and the decision is affected by multiple factors not incorporated into the testing performed here, the implants compared here did have different neck lengths and this could have affected our results. The 2 mm shorter neck length of the CFX implant could have resulted in a stiffer construct and higher torque experienced under the same applied force, compared to the paired BFX + lb constructs.

There was no difference between BFX + lb and CFX implanted femora when tested in 4-point bending. The median loads at ultimate failure were >4,000 N, and all constructs failed at >3,000 N. A prior study that implanted instrumented hip replacement prostheses and femoral cortical strain gauges into 25–35 kg dogs determined that the maximum net force on the femur was up to 1.65 times body weight during stance phase of the walking gait, which was roughly resolved into lateral tension, medial compression, and internal rotation (Page et al., 1993). In the current study, the limbs were obtained from medium to large breed dogs with an estimated average body weight of approximately 30 kg, or 294 N. Using this 1.65 multiplication factor, we can infer that the net force that the implant/bone constructs would be expected to withstand during walking would be 486 N in our average femur, which would be resolved into a variety of vectors, including medial-lateral bending as was applied in the current study. It stands to reason that the magnitude required for failure in this study (>3,000 N) in medial-lateral bending would be substantially higher than the *in vivo* forces that would be experienced by similarly sized dogs (even after resolution of weight bearing forces into bending and other forces). Therefore, we conclude that both BFX + lb and CFX implanted femora can withstand the lateral tensile and medial compressive forces experienced during walking, immediately after prosthesis implantation. Importantly, the bending test design used here did not include the proximal femur where the stem enters the bone and where the lateral bolt is incorporated into the construct and a different study design such as placing the bending force directly on the head of the implant may reveal entirely different conclusions.

Fracture configuration was not different between BFX + lb and CFX treatment groups in the torsional test based on the Vancouver classification, however the fracture mechanism may be different between treatment groups. Fracture occurs in BFX implanted femurs under torsional loads because the stem has an oval-shaped cross-section. As torsional force is applied to the implant head, the BFX stem rotates within the medullary cavity and the diameter of the implant will eventually exceed that of the bone in the sagittal plane. With the addition of the lateral bolt to the BFX stem, the lateral bolt was noted to contact the cranial border of the hole in the lateral cortex, and the presence of the hole likely acts as a stress riser. In contrast, as the CFX stem rotated, the rotation was translated through the cement to the cortical bone and the cortical bone was noted to twist with the implant to some degree. Despite the potential differences in mechanism of fracture, the fracture configuration was again similar between treatment groups, although the fracture did originate from the bolt hole in two BFX + lb implanted femurs. Three CFX stems were loose after torsional testing; the instability was noted to be at the stem-cement interface rather than the cement-bone interface. Loosening in clinical cases of aseptic loosening also primarily occurs at the stem-cement interface (Edwards et al., 1997; Skurla et al., 2005), which is likely related to the smooth interface between the stem and the cement as compared to the interdigitating interface between the cement and the bone. Clinically, failure at either interface could result in aseptic loosening. It should be noted that we were not always able to visualize fracture initiation and propagation completely since only a single camera angle was utilized, but the lateral aspect of the femur was always visualized during torsional testing to fully examine the interaction of the bolt and bolt hole during testing and the bones were thoroughly examined after testing as well.

Fracture configuration was also not different between BFX + lb and CFX treatment groups in the 4-point bending test. The 4-point bending test applies a uniform bending moment between the load (inside) rollers (ASTM International SDoMP, 2010), which allowed us to test a reported site of post-THR fracture at the distal aspect of the stem ( ± cement mantle) (Liska, 2004; Ganz et al., 2010; Monotti et al., 2020). Both BFX + lb and CFX implanted femora should have a stress riser at the distal aspect of the stem ( ± cement mantle) (Marcellin-Little et al., 2010; Small et al., 2017), and this was reflected in the resultant fracture configurations (11/12 were Vancouver class B1 or B2). Because the 4-point bending test does not apply a high bending force to the region of the femur containing the lateral bolt hole, no conclusion can be made as to whether the lateral bolt hole creates a stress riser under bending forces.

This study has many limitations that must be considered. A type 2 statistical error may be present and a larger sample size may have revealed more significant differences between treatment groups, especially with regards to torque at ultimate failure in torsion as only five femurs failed in each implant group resulting in four comparable pairs. According to *post hoc* analysis, at least seven paired failed samples would be needed to demonstrate a difference in torque at ultimate failure and we were unable to obtain additional paired samples to achieve this number. Additional clinically relevant tests could include axial loading through the head of the implant or a cyclic loading of walk, trot, gallop, or jumping forces to replicate daily activities–the acute load to failure test performed here does not replicate *in vivo* forces and therefore the results must be interpreted carefully. The 4-point bending test does not test the portion of the bone proximal or distal to the load (inside) rollers and therefore we were not able to draw conclusions about the effect of the presence of the lateral bolt and lateral bolt hole in the BFX + lb implanted femora. While both the 4-point bending and torsional tests were designed to apply a purely bending or torsional load, respectively, it is unlikely that we achieved this as the shape of the femur is not a straight column, each pair of bones is unique, each bone is a mirror image of the contralateral bone, and the potting and positioning technique was ultimately subjective despite every attempt at accuracy. There was a difference in version angle between implant groups tested in 4-point bending and there may be a type 2 statistical error preventing identification of a difference in version angle between groups tested in axial torsion. It is not clear exactly what effect this may have had on the results, though it should be noted that this is unlikely to have affected results for 4-point bending, and for axial torsion the femur was able to be loaded into the testing machine to accommodate for variations in version as the base attachment was able to rotate prior to clamping to the base of the machine. The BFX + lb and CFX stems are created using different metals (titanium and cobalt chrome, respectively) which have different biomechanical properties but because they are used interchangeably within the size ranges we reported this is not a limitation but rather a representation of the clinical situation. Importantly, the femurs in this study had CFIs between 1.81–2.24 (“normal” morphology, defined as a CFI 1.8–2.5), rather than stovepipe (CFI < 1.8) or other morphologies that would indicate that either a CFX or BFX + lb stem would be necessitated and therefore we cannot make conclusions regarding differences between these implant types in morphologies other than normal cadaveric femurs. Cadaver acquisition is increasingly challenging at our institution and during the entire collection period, no stovepipe femurs were obtained and we were unable to add paired femurs to increase sample size to meet a sample size that may have shown more statistical significance as implied by post-hoc analysis. It may be that a model to replicate stovepipe morphology and provide higher sample numbers, such as a 3D printed model, may prove to be relevant for studies such as this one. Lastly, these results can only be extrapolated to the immediately post-operative clinical scenario at most. For example, it is not known whether a BFX implant with reduced stiffness could be experiencing micromotion leading to loosening over time, and typically osteointegration will occur and strengthen BFX constructs and loosening will occur and weaken CFX constructs over time, as well as complex bone resorption and formation after either cemented or uncemented total hip replacement; all of these temporal variables will change the stiffness and failure parameters.

In conclusion, CFX implanted normal cadaveric femurs were shown to have higher stiffness than BFX + lb implanted normal cadaveric femurs when tested in torsion and both CFX and BFX + lb implanted femurs failed at loads higher than experienced *in vivo*. While we suspect that both constructs should withstand *in vivo* forces despite the difference in stiffness, due to the differences between acute load to failure test and the cyclic loading experienced *in vivo*, we cannot definitively conclude whether this statistical difference is clinically relevant. Based on this study, the BFX + lb system is suspected to be interchangeable with the CFX system when applied to normal femora, although additional bending tests to incorporate the entire proximal femur and lateral bolt hole, axial compression tests, and torsional testing (higher sample numbers, higher displacement) could also be performed, both to failure and non-destructive cyclic testing, for further evidence. Additionally, further testing would need to be performed in order to ascertain whether this conclusion remains true and whether other differences are identified when these methods are applied to femurs with stovepipe morphology as this morphology is one of the morphologies where the BFX + lb stem is promoted for use but not yet proven to be safe.

## Data Availability

The raw data supporting the conclusion of this article will be made available by the authors, without undue reservation.

## References

[B1] ASTM International SDoMP (2010). Book of standards. Standard test method for flexural properties of unreinforced and reinforced plastics and electrical insulating materials by four point bending. West Conshohocken, PA: ASTM International, 9.

[B2] Barrack R. L. Mulroy R. D. Harris W. H. (1992). Improved cementing techniques and femoral component loosening in young patients with hip arthroplasty: A 12-year radiographic review. J. Bone Jt. Surg. Br. 74-B (3), 385–389. 10.1302/0301-620x.74b3.1587883 1587883

[B3] Bausman J. Wendelburg K. (2013). Femoral prosthesis version angle calculation from a sagittal plane radiographic projection of the femur. Vet. Surg. 42, 398–405. 10.1111/j.1532-950x.2012.01078.x 23240574

[B4] Bergh M. S. Gilley R. S. Shofer F. S. Kapatkin A. S. (2006). Complications and radiographic findings following cemented total hip replacement: A retrospective evaluation of 97 dogs. Vet. Comp. Orthop. Traumatol. 19, 172–179. 10.1055/s-0038-1632994 16972000

[B5] Brady O. Garbuz D. Masri B. Duncan C. (2000). The reliability of validity of the Vancouver classification of femoral fractures after hip replacement. J. Arthroplasty 15, 59–62. 10.1016/s0883-5403(00)91181-1 10654463

[B6] Buks Y. Wendelburg K. L. Stover S. M. Garcia-Nolan T. C. (2016). The effects of interlocking a universal hip cementless stem on implant subsidence and mechanical properties of cadaveric canine femora. Vet. Surg. 45, 155–164. 10.1111/vsu.12437 26767439 PMC5066748

[B7] Christopher S. A. Kim S. E. Roe S. Pozzi A. (2016). Biomechanical evaluation of adjunctive cerclage wire fixation for the prevention of periprosthetic femur fractures using cementless press-fit total hip replacement. Vet. J. 214, 7–9. 10.1016/j.tvjl.2016.04.014 27387718

[B8] DeYoung D. J. Schiller R. A. (1992). Radiographic criteria for evaluation of uncemented total hip replacement in dogs. Vet. Surg. 21 (2), 88–98. 10.1111/j.1532-950x.1992.tb00021.x 1626388

[B9] Edwards M. R. Egger E. L. Schwarz P. D. (1997). Aseptic loosening of the femoral implant after cemented total hip arthroplasty in dogs: 11 cases in 10 dogs (1991-1995). J. Am. Vet. Med. Assoc. 211 (5), 580–586. 9290824

[B10] Engh C. A. O'Connor D. Jasty M. McGovern T. F. Bobyn J. D. Harris W. H. (1992). Quantification of implant micromotion, strain shielding, and bone resorption with porous-coated anatomic medullary locking femoral prostheses. Clin. Orthop.Relat. Res. 285, 13–29. 10.1097/00003086-199212000-00005 1446429

[B11] Ganz S. M. Jackson J. VanEnkevort B. (2010). Risk factors for femoral fracture after canine press-fit cementless total hip arthroplasty. Vet. Surg. 39, 688–695. 10.1111/j.1532-950X.2010.00694.x 20459487

[B12] Hoefle W. D. (1974). A surgical procedure for prosthetic total hip replacement in the dog. J. Am. Anim. Hosp. Assoc. 10 (3), 269–276.

[B13] Johnson J. Austin C. Breur G. (1994). Incidence of canine appendicular musculoskeletal disorders in 16 veterinary teaching hospitals from 1980 through 1989. Vet. Comp. Orthop. Traumatol. 7 (2), 56–69. 10.1055/s-0038-1633097

[B14] Kowaleski M. (2013). “Biomechanical considerations in total hip replacement,” in Advances in small animal total joint replacement. Editors Peck J. Marcellin-Little D. (Ames, Iowa: Wiley-Blackwell), 53–68.

[B15] Liska W. D. (2004). Femur fractures associated with canine total hip replacement. Vet. Surg. 33, 164–172. 10.1111/j.1532-950x.2004.04024.x 15027978

[B16] Marcellin-Little D. J. Cansizoglu O. Harrysson O. L. A. Roe S. C. (2010). *In vitro* evaluation of a low-modulus mesh canine prosthetic hip stem. Am. J. Vet. Res. 71 (9), 1089–1095. 10.2460/ajvr.71.9.1089 20807150

[B17] Marcellin-Little D. J. DeYoung B. A. Doyens D. H. DeYoung D. J. (1999). Canine uncemented porous-coated anatomic total hip arthroplasty: Results of a long-term prospective evaluation of 50 consecutive cases. Vet. Surg. 28, 10–20. 10.1053/jvet.1999.0010 10025635

[B18] Mitchell M. Hudson C. Beale B. (2020). Comparison of femoral stem subsidence between three types of press-fit cementless total hip replacement in dogs. Vet. Surg. 49, 787–793. 10.1111/vsu.13391 32086832

[B19] Monotti I. C. Preston C. A. Kidd S. W. (2020). Treatment outcomes for periprosthetic femoral fractures in cementless press-fit total hip replacement. Vet. Comp. Orthop. Traumatol. 33, 370–376. 10.1055/s-0040-1709486 32356296

[B20] Olmstead M. L. Hohn R. B. Turner T. M. (1983). A five-year study of 221 total hip replacements in the dog. J. Am. Vet. Med. Assoc. 183 (2), 191–194. 6885591

[B21] Olmstead M. L. (1987). Total hip replacement. Vet. Clin. North Am. Small Anim. Pract. 17 (4), 943–955. 10.1016/s0195-5616(87)50086-9 3303636

[B22] Ota J. Cook J. L. Lewis D. D. Tomlinson J. L. Fox D. B. Cook C. R. (2005). Short-term aseptic loosening of the femoral component in canine total hip replacement: Effects of cementing technique on cement mantle grade. Vet. Surg. 34, 345–352. 10.1111/j.1532-950x.2005.00053.x 16212589

[B23] Page A. E. Allan C. Jasty M. Harrigan T. P. Bragdon C. R. Harris W. H. (1993). Determination of loading parameters in the canine hip *in vivo* . J. Biomech. 26 (4/5), 571–579. 10.1016/0021-9290(93)90018-a 8478358

[B24] Pozzi A. Peck J. N. Chao P. Choate C. J. Barousse D. Conrad B. (2013). Mechanical evaluation of adjunctive fixation for prevention of periprosthetic femur fracture with the Zurich cementless total hip prosthesis. Vet. Surg. 42, 529–534. 10.1111/j.1532-950x.2013.12018.x 23731463

[B25] Rashmir-Raven A. M. DeYoung D. J. Abrams C. F. Aberman H. A. Richardson D. C. (1992). Subsidence of an uncemented canine femoral stem. Vet. Surg. 21 (5), 327–331. 10.1111/j.1532-950x.1992.tb01705.x 1413463

[B26] Schiller T. D. (2017). BioMedtrix total hip replacement systems: An overview. Vet. Clin. North Am. Small Anim. Pract. 47, 899–916. 10.1016/j.cvsm.2017.03.005 28576274

[B27] Skurla C. P. Pluhar G. E. Frankel D. J. Egger E. L. James S. P. (2005). Assessing the dog as a model for human total hip replacement: Analysis of 38 canine cemented femoral components retrieved at post-mortem. J. Bone.Joint Surg. Br. 87-B (1), 120–127. 10.1302/0301-620x.87b1.14678 15686252

[B28] Small S. R. Hensley S. E. Cook P. L. Stevens R. A. Rogge R. D. Meding J. B. (2017). Characterization of femoral component initial stability and cortical strain in a reduced stem-length design. J. Arthroplasty 32, 601–609. 10.1016/j.arth.2016.07.033 27597431

[B29] Townsend S. Kim S. E. Pozzi A. (2016). Effect of stem sizing and position on short-term complications with canine press fit cementless total hip arthroplasty. Vet. Surg. 46, 803–811. 10.1111/vsu.12666 28460422

